# Effect of Presence of Uni- or Bilateral Thyroid Adenoma on Recovery of Pituitary–Thyroid Axis and Creatinine Concentration in Radioiodine-Treated Cats

**DOI:** 10.3390/ani14182627

**Published:** 2024-09-10

**Authors:** Anna Paulina Menzel, Joanna Lin, Arne Güssow, Ve Patzelt, Natali Bauer, Katarina Hazuchova

**Affiliations:** Clinic for Small Animals (Internal Medicine, Clinical Pathology and Clinical Pathophysiology), Justus-Liebig University of Giessen, 35392 Giessen, Germanykatarina.hazuchova@vetmed.uni-giessen.de (K.H.)

**Keywords:** thyroxine, thyroid-stimulating hormone, iatrogenic hypothyroidism, azotaemia, chronic kidney disease, radioiodine treatment, feline hyperthyroidism

## Abstract

**Simple Summary:**

Hyperthyroidism is the most common endocrinopathy in older cats and radioiodine therapy (RAIT) is the gold standard for its treatment. Nevertheless, information about changes in thyroid and kidney values after RAIT over a period longer than six months and the effect of the presence of uni- or bilateral thyroid adenoma on these values is scarce or unreported. In our study, we evaluated thyroid and kidney values over a period of 6 to 12 months following RAIT. Total thyroxine (TT4) normalised within a month of treatment in most cats, but hypothyroidism or an increase in creatinine concentration indicating kidney disease could be diagnosed at any timepoint between 1 and 12 months post RAIT, although in most cats, these conditions were diagnosed within 6 months of treatment. Furthermore, cats with bilateral adenoma had significantly higher thyroid-stimulating hormone (TSH) concentrations after RAIT compared to those with unilateral adenoma. Our study highlights the importance of the ongoing revaluation of cats post RAIT in order to screen for patients in which treatment resulted in iatrogenic hypothyroidism or unmasked chronic kidney disease, with both conditions necessitating treatment to improve outcomes.

**Abstract:**

Radioiodine therapy (RAIT) is the gold standard for treatment of hyperthyroidism in cats. The aim of this study was to evaluate the effect of the presence of uni- or bilateral thyroid adenoma on changes in total thyroxine (TT4), thyroid-stimulating hormone (TSH), and creatinine concentration over a period of 6 to 12 months following RAIT. Fifty-one hyperthyroid cats presented for RAIT between April 2021 and April 2022 were prospectively enrolled. Cats with an increased creatinine concentration (creatinine ≥ 140 µmol/L), renal morphology abnormalities, and suspected thyroid carcinoma were excluded. TT4, TSH, and creatinine were determined before and one week and one, three, six, and twelve months following RAIT. The effects of the re-examination timepoint following RAIT and the presence of uni- or bilateral thyroid adenoma based on technetium-99m scintigraphy on TT4, TSH, and creatinine were analysed by mixed effects modelling. Cats with bilateral adenoma had significantly higher TSH concentrations after RAIT compared to those with unilateral adenoma. TT4 concentration significantly decreased one week (*p* < 0.001) and again one month following RAIT (*p* < 0.001). TSH and creatinine concentration significantly increased one month post RAIT (both *p* < 0.001). As indicated by an increase in TSH concentration, the pituitary–thyroid axis needs a minimum of one month post RAIT to recover from hyperthyroidism-induced suppression, but hypothyroidism necessitating levothyroxine supplementation might not be diagnosed before 6 or even 12 months post RAIT. Although creatinine did not increase significantly after one month post RAIT in this cohort, an increased creatinine concentration was detected at later timepoints in individual cats.

## 1. Introduction

With a prevalence of 3.93–20.14%, hyperthyroidism is the most common endocrinopathy in the population of older cats [1,2,3,4,5]. Of all options, radioiodine therapy (RAIT) is considered the gold standard for treatment of hyperthyroidism due to its high success rate and minimal invasiveness. In most cats, concentrations of total thyroxine (TT4) decline into the reference range (RR) within two weeks after RAIT [6]. However, a proportion of cats might develop iatrogenic hypothyroidism following therapy. This condition might not be recognised if only TT4 is measured to assess the success of RAIT. A recent study suggested that thyroid-stimulating hormone (TSH) is a better parameter than TT4 to identify iatrogenic hypothyroidism when compared to the gold standard evaluation of thyroid function by scintigraphy [7]. In hyperthyroid cats, TSH concentration is usually undetectable due to the suppression of the pituitary–thyroid axis secondary to high TT4 concentration [8]. In the long term following the resolution of hyperthyroidism, TSH is expected to normalise in cats that achieve euthyroidism, or increase above the RR in cats that develop hypothyroidism. Although TSH measurement has been integrated into follow-up evaluations of cats post RAIT, most studies merely use TSH to identify hypothyroid cats but do not provide more detail on changes in TSH concentration over time or optimal time for its measurement following RAIT.

Besides TT4 and TSH, another parameter to monitor after RAIT is creatinine (or symmetric dimethylarginine [SDMA]) as a marker of kidney function. Studies have shown that the glomerular filtration rate (GFR) decreased one month following RAIT [9,10,11], which led to development of azotaemia in 10/24 (42%) cats followed-up for six months after RAIT in one study [10]. Other larger studies reported that 16–33% of cats became azotaemic following RAIT [12,13,14]. These latter studies [12,13,14], however, used their own laboratory RR to establish the diagnosis of azotaemic chronic kidney disease (CKD), with creatinine cut-offs considerably higher than the cut-off ≥ 140 µmol/L recommended by the International Renal Interest Society (IRIS) [15]. In RAI-treated cats, the early identification of azotaemia might be particularly important in those that develop hypothyroidism, because the restoration of euthyroidism (by providing levothyroxine (LT4) supplementation) might lead to the improvement or resolution of azotaemia and improve survival [7]. Because most studies follow RAI-treated cats for only six months [12,13,14], it is largely unreported if any cats become hypothyroid and azotaemic following this period and could potentially benefit from treatment. In two studies that evaluated cats at 6 to 12 months post RAIT, it was not specified what proportion of cats was followed-up for 12 months and if any cats became hypothyroid or azotaemic later than 6 months of therapy [16,17].

Furthermore, to the authors’ knowledge, the impacts of the presence of uni- or bilateral thyroid adenoma on changes in TT4, TSH, and creatinine concentration following RAIT have not been systematically assessed. The number of foci detected on thyroid scintigraphy and the presence of bilateral disease have been associated with low TT4 and iatrogenic hypothyroidism after RAIT, respectively [18,19]. However, these studies focused on association with outcome rather than longitudinal changes in the thyroid values or creatinine. It could be hypothesised that the pituitary–thyroid axis might be more severely suppressed in cats with bilateral adenoma when compared to cats with unilateral disease and therefore might need longer time to recover (i.e., increase in TSH concentration might be delayed in cats with bilateral disease).

The aims of this study were therefore threefold: (1) to collect data on the recovery of the pituitary–thyroid axis following RAIT (i.e., timepoint of significant increase in TSH concentration) and describe changes in TSH concentration over a period of 6 to 12 months after RAIT; (2) to assess if any cats become hypothyroid and experience an increase in creatinine concentration 6 to 12 months following RAIT; (3) to evaluate the effect of the presence of uni- or bilateral thyroid adenoma on changes in TT4, TSH, and creatinine concentration over a period of 6 to 12 months after RAIT.

## 2. Materials and Methods

This study describes longitudinal changes in TT4, TSH, and creatinine concentration in hyperthyroid cats treated with RAIT over a period of 6 to 12 months, with the main focus on the recovery of the pituitary–thyroid axis and the effect of the presence of uni- or bilateral thyroid adenoma on these values. Hyperthyroid cats presented for RAIT at the Small Animal Clinic, Department of Internal Medicine of Justus-Liebig-University (JLU) Giessen, Germany, between April 2021 and April 2022 were prospectively enrolled. A diagnosis of hyperthyroidism was made by the referring veterinarians based on the clinical signs (e.g., weight loss, polyphagia, behavioural changes, polyuria/polydipsia, vomiting, diarrhoea) and increased TT4 (using their own laboratory RR). Cats with an increased creatinine concentration (creatinine ≥ 140 µmol/L) [15] upon presentation for RAIT were excluded. Because cats also participated in another study evaluating renal morphology as a predictor for development of azotaemic CKD post RAIT, cats with renal morphological abnormalities (e.g., renal cysts) preventing participation in that study were also excluded. There were no other exclusion criteria. All owners signed an informed consent upon enrolment. This study was approved by the local ethical committee and registered by the regional authority (Regierungspräsidium Giessen, 19 c 20 15 h 02 Gi 18/17 kTV 14/2021).

### 2.1. Screening Tests

Detailed history (including previous treatment) as well as thorough physical examinations were performed upon admission for RAIT. Cats underwent screening tests including complete blood cell count (ADVIA 2120i, Siemens Healthineers, Erlangen, Germany), heparin plasma biochemistry including measurement of creatinine (ABX Pentra 400, HORIBA ABX SAS, Montpellier, France), urinalysis (all three performed at the Central Laboratory of the Small Animal Clinic, Giessen, Germany), TT4 and TSH measurement, thoracic radiographs, echocardiography, blood pressure measurement, and ultrasound of the urinary tract (as a part of another study) before treatment. TT4 and TSH were measured at an external laboratory (Biocontrol, Ingelheim, Germany) using a chemiluminescence assay for measurement of feline TT4 (Immulite 2000, Siemens Healthineers, Erlangen, Germany) and a chemiluminescence assay for measurement of human TSH (ADVIA Centaur, Siemens Healthineers, Erlangen, Germany; intra-assay coefficient of variation [CV]: 4.3%, inter-assay CV: 2.7%; further information provided in Supplementary Materials) [20], which was used in previous studies [21,22,23]. The laboratory Biocontrol participates in the European Veterinary Endocrine Quality Assurance Scheme and the TSH assay passed the quality control criteria (see Supplementary Materials). The reference range for TT4 was 1–4 μg/dL (12.9–51.5 nmol/L), with a lower limit of detection of 0.5 μg/dL (6.4 nmol/L), and the RR for TSH was 0.04–0.44 µU/mL, with a lower limit of detection of 0.015 µU/mL [20]. Both RRs were determined by the external laboratory. Cats with TT4 < 13 μg/dL (<167.3 nmol/L) were considered to have mild-to-moderate hyperthyroidism, in agreement with a previous study [12].

### 2.2. Scintigraphy and RAIT

Antithyroid medication and a low-iodine diet were discontinued at least 7 and 14 days prior to RAIT, respectively. Prior to RAIT, scintigraphy was performed approximately 20–40 min after an intravenous injection of 30–40 MBq technetium-99m (Tc). Planar whole body and zoom images (to capture thyroid and salivary glands) were acquired for five minutes on a 256 × 256 matrix using a single-head gamma camera equipped with a low-energy-high-resolution (LEHR) collimator (DIACAM^®^, MiE GmbH, Seth, Germany). For scintigraphy, cats were sedated with 5 mg/kg ketamine (Ketamin at 100 mg/kg, CP Pharma, Burgdorf, Germany) and 0.5 mg/kg diazepam (Diazedor at 5 mg/kg, WDT, Garbsen, Germany) and positioned in sternal recumbency. Using scintigraphy findings, thyroid disease was described as either unilateral adenoma, bilateral adenoma, or suspected carcinoma [24]. Unilateral adenoma described the involvement of a single thyroid lobe, with the contralateral lobe not visualised. In cats with bilateral adenoma, both thyroid lobes were involved and visible. There was a single cat with bilateral adenoma and a small third nodule, which for the purpose of the statistical analysis in this study was included among cases of bilateral adenoma. Thyroid carcinoma was suspected where a huge volume of hyperfunctional thyroid tissue with several/multiple areas of increased radionuclide uptake was present. Moreover, the pattern of radionuclide uptake was usually heterogenous and the tumour margins irregular. An individualised dose of radioiodine was based on the percent uptake of technetium (TcTU), with cats with TcTU < 4% receiving a dose of 1–3 mCi (37–111 MBq), and cats with TcTU > 4% receiving 4–6 mCi (148–222 MBq). Furthermore, TT4 concentration (similar ranges to a previously published scheme) [25], and the presence of uni- or bilateral disease (lower dosages in cats with bilateral disease) were used to refine the dose within these two dose intervals based on TcTU. Cats with suspected carcinoma were treated with 10–30 mCi (370–1110 MBq) radioiodine. Radioiodine was injected intravenously. All scintigraphies and treatments were performed by a single clinician (N.B.).

### 2.3. Re-Examinations and Treatment Outcome

The first re-examination took place during hospitalisation five days following RAIT (week 1), while re-examinations one, three, six, and twelve months after RAIT were performed at cats’ local veterinary practices. Blood samples were taken by local veterinarians and centrifuged and heparin plasma as well as serum were sent by post to the Small Animal Clinic of JLU Giessen. On all occasions, biochemistry (including creatinine measurement) was performed at the Central Laboratory (see above), and measurements of TT4 and TSH were performed at an external laboratory (Biocontrol).

The outcome was defined as the resolution of hyperthyroidism if the cat became eu- or hypothyroid, or treatment failure if the cat remained hyperthyroid post RAIT. The diagnosis of persistent hyperthyroidism (TT4 > 4 µg/dL) was made no earlier than six months after RAIT, but if cats were symptomatic for their hyperthyroidism, they were started on antithyroid drugs prior to this timepoint and the medication was discontinued one week prior to the re-examinations (and re-started when hyperthyroidism was re-confirmed). Although the resolution of hyperthyroidism could be established from a single TT4 measurement within or below the RR post RAIT (if only a single follow-up TT4 measurement was available), the classification of the outcome into euthyroid (TT4: 1–4 µg/dL, TSH < 0.44 µU/mL) or hypothyroid (subclinical hypothyroidism: TT4 < 2.5 µg/dL and TSH > 0.44 µU/mL; overt hypothyroidism: TT4 < 1 µg/dL and TSH > 0.44 µU/mL) was made the earliest at month 6 post RAIT, or, if available, at month 12 post RAIT [26]. The only exception was the establishment of the need for LT4 replacement (i.e., hypothyroidism requiring LT4 replacement), which could be made before the month-6 re-examination if creatinine ≥ 140 µmol/L was detected for the first time in a cat with TT4/TSH indicating overt or subclinical hypothyroidism, or in a cat with overt hypothyroidism and corresponding clinical signs (e.g., lethargy, weight gain, poor coat quality). LT4 treatment was started at a dose of 100 µg/cat once daily (ideally given in the evening) and the dose was increased by 50 µg every four weeks until a TT4 concentration of 2.5–3.5 µg/dL was achieved [26]. At these re-examinations, TT4 and TSH were checked approximately 12 h following LT4 administration (usually in the morning).

An increased creatinine concentration was defined as creatinine ≥ 140 µmol/L, using the cut-off for IRIS stage 2 CKD [15] rather than a laboratory-own cut-off (RR for creatinine concentration at our laboratory: 0–168 µmol/L). This definition of an increased creatinine concentration is used in all RAI-treated cats at our institution as a part of routine management to allow for standardised treatment interventions (i.e., institution of LT4 replacement in hypothyroid cats with newly detected creatinine ≥ 140 µmol/L) when cats are followed-up at different veterinary practices, using different methods for creatinine measurement (and therefore different creatinine cut-offs). Therefore, a creatinine cut-off of 140 µmol/L was also adopted in the present study. The diagnosis of CKD (and treatment recommendations according to IRIS, e.g., feeding renal diet), however, was only made in cats with a persistent elevation of creatinine ≥ 140 µmol/L (i.e., only in cats with a minimum follow-up of six months post RAIT). Creatinine > 140 µmol/L (in conjunction with urine specific gravity [USG] < 1035 and evidence of persistence of laboratory findings or clinical signs of CKD) has been suggested by the International Society of Feline Medicine (ISFM) guidelines to make a diagnosis of CKD [27].

In cats with a newly detected increase in creatinine ≥ 140 µmol/L that had a follow-up of at least 6 or 12 months following RAIT, it was documented whether creatinine remained increased or dropped below 140 µmol/L during the follow-up.

### 2.4. Statistical Analysis

Data were tested for normality and because most data were not normally distributed, they are reported as the median (range, interquartile range [IQR]). Mixed effects modelling was performed to assess the effects of the presence of uni- or bilateral thyroid adenoma and study timepoint (enrolment and week 1, month 1, month 3, month 6, and month 12 following RAIT) on TT4, TSH, and creatinine concentration. To meet the assumptions of the model, TT4 and TSH were transformed using log (TT4 + 1) and √TSH, respectively. Fisher’s least significant difference (LSD) tests were used for post hoc comparisons when factors showed a significant effect on the outcome variable. Cats with suspected carcinoma as well as cats with persistent hyperthyroidism were excluded from this analysis. In addition, test results of cats with hypothyroidism from re-examination timepoints after starting LT4 replacement were excluded from the analysis of TT4 and TSH.

To assess whether any variables were associated with a TSH increase above the RR, categorical (proportion of cats with uni- or bilateral disease) and numerical (TT4 concentration prior to RAIT, radioiodine dose) variables were compared between cats that experienced a TSH increase >RR and those that did not using Fisher’s exact test and the Mann–Whitney U test, respectively. Only cats with uni- or bilateral adenoma and the resolution of hyperthyroidism and a minimum of a one-month follow-up were included in the analysis.

The statistical analysis was performed using SPSS ver. 28 (IBM Statistics, Armonk, NY, USA) and graphs were made using GraphPad Prism ver. 9 (GraphPad Software, Boston, MA, USA).

## 3. Results

Eighty-one cats presented for RAIT during the study period, and fifty-one cats were enrolled. However, because in one cat no re-checks were performed following RAIT, this cat was excluded due to the unknown outcome. The reasons for the remaining 30/81 cats for not being enrolled into this study are in the Supplementary Materials.

The median age of the 50 included cats was 12 years (range: 7–18, IQR: 10–13). Thirty-two cats were neutered females and eighteen were neutered males. There were thirty-eight Domestic Shorthair, three Norwegian Forest, two Maine Coon, two mixed-breed, one Persian, one Chartreux, one British Shorthair, one Russian Blue, and one Birman cat.

Forty-eight cats received treatment for hyperthyroidism prior to presentation (antithyroid drugs: n = 42; low-iodine diet: n = 6). Out of the six cats that were treated with a low-iodine diet, five received antithyroid drugs before being switched to the diet. In 22/50 (44%), hyperthyroidism was considered mild to moderate based on pre-treatment TT4 concentration < 13 μg/dL. Scintigraphy findings indicated unilateral adenoma in 19 cats, bilateral adenoma in 26 cats, and a single cat had a third small adenoma (this cat was grouped together with the bilateral adenomas for the purpose of the statistical analysis). In four cats, carcinoma was suspected based on scintigraphy. Information about TcTU, homogeneity of TcTU, and thyroid volume is provided in the Supplementary Materials. The 50 cats were treated with a median iodine dose of 4.2 mCi (range: 1.5–25, IQR: 2.6–5).

The four cats with suspected carcinoma were excluded from the statistical analysis because cats with suspected carcinoma are treated differently to cats with thyroid adenomas (higher iodine dose is needed to achieve resolution of hyperthyroidism). Therefore, in the following sections, only the 46 cats with adenomas are described.

### 3.1. Follow-Up and Treatment Outcome

A total of 4 cats (4/46, 9%) with adenomas remained hyperthyroid post RAIT. Three of the four cats that remained hyperthyroid were started on antithyroid medication and one received a low-iodine diet. These cats were not included in any further statistical analysis.

In the remaining 42 cats (42/46, 91%), the resolution of hyperthyroidism was confirmed by at least one TT4 measurement post RAIT within or below the RR. These 42 cats underwent a median of 4 (range: 1–5, IQR: 3–5) of the 5 scheduled re-examinations as a part of this study. The median (range, IQR) concentration of TT4, TSH, and creatinine at each study timepoint and the number of cats for which these test results were available can be found in Table 1.

**Figure 1:**
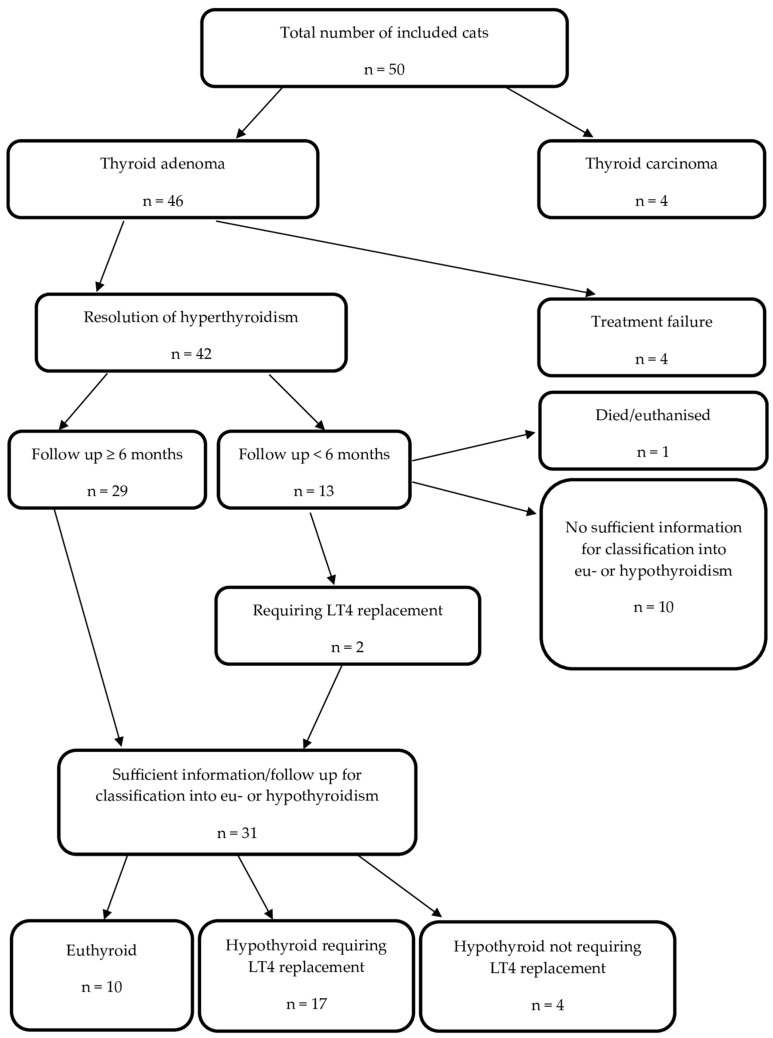
Treatment outcome in 50 cats treated with radioactive iodine between April 2021 and April 2022. Legend: n, number of cats; LT4, levothyroxine.

**Figure 2:**
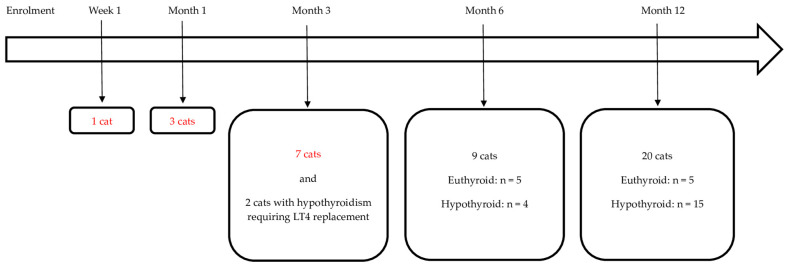
The final visit and thyroid status (if follow-up of at least 6 months was available) in the 42 cats with uni- or bilateral adenoma and the resolution of hyperthyroidism following radioiodine treatment. Of these 42 cats, 10 became euthyroid and 21 became hypothyroid (17/21 required levothyroxine (LT4) treatment) and in 11 cats, the follow-up was <6 months and not sufficient to differentiate between eu- and hypothyroidism (these cats with insufficient follow-up are marked in red). Two of the twenty-one hypothyroid cats left this study following month-3 re-examination but their outcome could be classified because they required LT4 replacement at month 1. Legend: n, number of cats.

The overview of the available follow-up and treatment outcome in the 50 cats that were included in this study and the 42 cats with uni- or bilateral adenoma and the resolution of hyperthyroidism is in Figure 1 and Figure 2, respectively.

**Figure 3:**
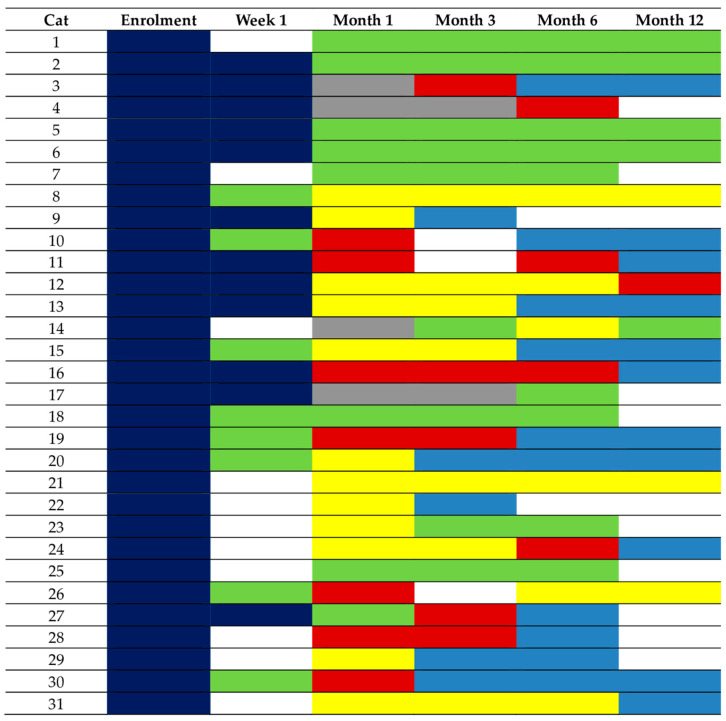
Changes in thyroid status depicted by different colours in the 31 cats with sufficient information to differentiate between eu- and hypothyroidism. These cats had a follow-up of 6 (n = 9) or 12 (n = 20) months following radioiodine treatment (RAIT), or hypothyroidism requiring levothyroxine (LT4) replacement was diagnosed before month 6 (cat 9 and cat 22, both started on LT4 replacement following month-1 re-examination). Of the 10 euthyroid cats, only 2 (cat 14 and cat 23) experienced transient hypothyroidism before becoming euthyroid. Legend: dark blue, hyperthyroidism; red, overt hypothyroidism; yellow, subclinical hypothyroidism; bright blue, levothyroxine (LT4) replacement; green, euthyroidism; grey, total thyroxine below the reference range and thyroid-stimulation hormone within the reference range (unclassifiable).

A total of 13 cats (13/42, 31%) were followed-up for <6 months. One of these cats was euthanised and insufficient data were available for the further classification of 10 cases. The remaining two cases were diagnosed as hypothyroid requiring LT4 replacement at month 1 post RAIT. Twenty-nine cats were followed-up for 6 (n = 9) or 12 months (n = 20). Consequently, 31/42 (74%) cats had sufficient information to differentiate between eu- and hypothyroidism. The changes in their thyroid status during this study are depicted in Figure 3. At their final study visit, 10 were euthyroid and 21 were hypothyroid. Of the 10 cats that became euthyroid, only 2 cats experienced transient hypothyroidism, which was subclinical in both cases (cat 14 at month 6 and cat 23 at month 1; see also Figure 3).

Of the 21 cats (21/50, 42%) that became hypothyroid following RAIT, 17 (17/21, 81%) required LT4 replacement (9/17 had overt and 8/17 had subclinical hypothyroidism at the time of starting LT4 replacement), and 4 cats (4/21, 19%) did not (2/4 with subclinical and 2/4 with overt hypothyroidism at their final study visit).

In the 17 hypothyroid cats requiring LT4 replacement, the diagnosis was made one month following RAIT in 6 cats (6/17), after three months in 6 cats (6/17), after six months in 4 cats (4/17), and after twelve months in 1 cat (1/17). However, most of these cats had low TT4 and/or increased TSH at one or two re-examinations prior to starting LT4 replacement (see Figure 3). In all 17 cats, LT4 supplementation was started after the detection of a new onset of an increased creatinine concentration (creatinine ≥ 140 µmol/L). Therefore, no cat was started on LT4 replacement due to clinical signs of hypothyroidism.

#### 3.1.1. The Presence of Uni- or Bilateral Disease

The number (proportion) of cats with uni-/bilateral adenoma that became euthyroid, became hypothyroid (including cats with and without LT4 replacement), or did not have a sufficient follow-up to differentiate between euthyroidism and hypothyroidism is summarised in Table 2.

#### 3.1.2. Occurrence of Increased Creatinine Concentration during Study

An increased creatinine concentration (creatinine ≥ 140 µmol/L) was detected at some point during this study in 28/42 (67%) of cats with uni- or bilateral adenoma and the resolution of hyperthyroidism. No cat had an increased creatinine concentration at week 1, but an increased creatinine concentration was detected in 11/37 (30%), 16/35 (46%), 13/28 (46%), and 10/20 (50%) cats for which creatinine measurement was available at month 1, month 3, month 6, and month 12, respectively. Twenty-two out of the twenty-eight cats with an increased creatinine concentration had a follow-up of at least 6 months post RAIT, and creatinine concentration dropped below 140 µmol/L in 10/22 (46%) of these cats. In 1 additional cat (1/22), creatinine increased above 140 µmol/L at the 12-month re-examination; therefore, it is not known whether the creatinine concentration dropped below 140 µmol/L afterwards. Of those 22 cats with an increased creatinine concentration and sufficient follow-up, 15 (68%) were hypothyroid and receiving LT4 replacement, and in 8/15 (53%) cats, creatinine concentration dropped below 140 µmol/L after starting LT4 replacement, while in 6/15 (40%), creatinine concentration remained increased (and in the 1 previously mentioned cat diagnosed with hypothyroidism requiring LT4 replacement at month 12, it is unknown whether creatinine concentration dropped below 140 µmol/L). Among the 10 cats that achieved euthyroidism post RAIT, 5/10 (50%) developed an increase in creatinine concentration but in 2/5, it was very mild (creatinine concentration between 140 and 150 μmol/L).

At the final follow-up 6 or 12 months post RAIT, in 11 cats (11/42, 26%) with the resolution of hyperthyroidism, a persistently increased creatinine concentration was documented; in all 11 cats within IRIS stage 2 (140–249 μmol/L) [15]. These cats were diagnosed with CKD.

### 3.2. Assessment of the Effects of the Presence of Uni- or Bilateral Thyroid Adenoma and Study Timepoint on TT4, TSH, and Creatinine Concentration

Results of 42 cats with uni- or bilateral thyroid adenoma and the resolution of hyperthyroidism were included in this analysis. However, in the 17 hypothyroid cats (17/42, 40%) that required LT4 replacement, the TT4 and TSH results of their re-examinations following the LT4 treatment start were removed from the analysis of the effects of the presence of uni- or bilateral thyroid adenoma and study timepoint on TT4 and TSH concentration. Table 3 shows the median (range, IQR) of TT4 and TSH concentration at each study timepoint as well as the number of cats, for which test results could be included in the statistical analysis.

**Figure 4:**
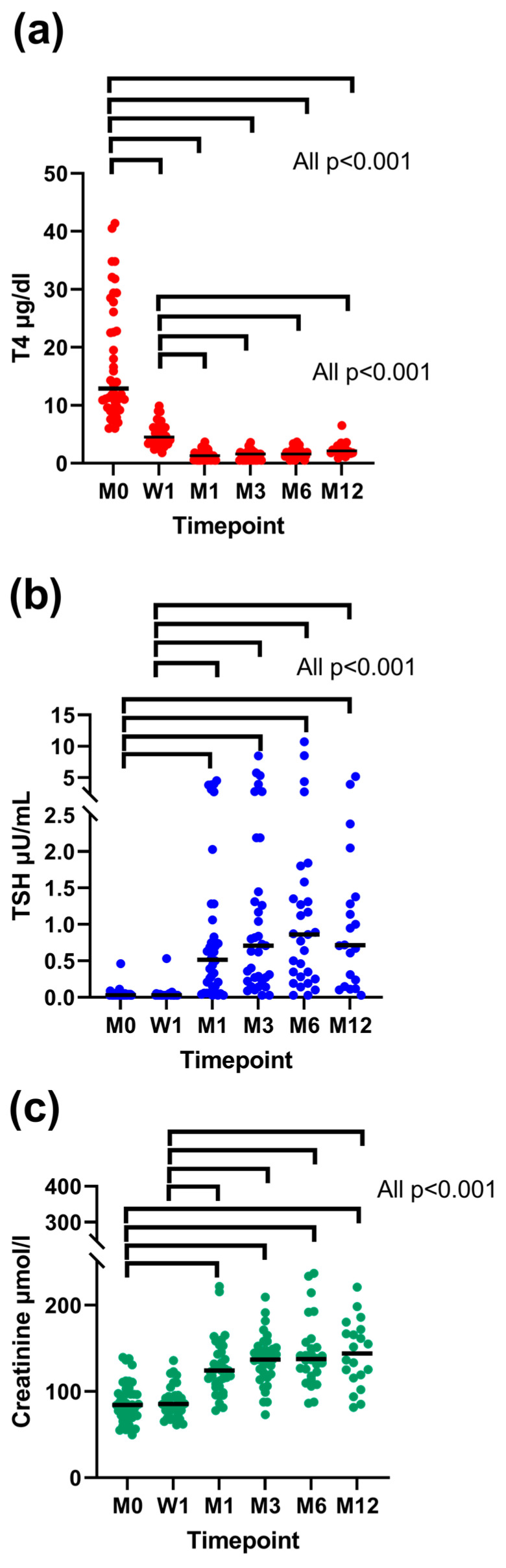
Concentrations of total thyroxine (TT4) (**a**), thyrotropin (TSH) (**b**), and creatinine (**c**) before and one week, one month, three months, six months, and twelve months after radioiodine treatment (RAIT). Only cats with uni-/bilateral adenoma and the resolution of hyperthyroidism were included. In cats receiving levothyroxine (LT4) replacement, the TT4 and TSH results from re-examinations following the LT4 treatment start were excluded from the analysis. Each dot represents the value of a single cat. Medians are presented as horizontal black lines. The significant differences in values between each timepoint are indicated by horizontal brackets. Legend: M, month; W, week.

The results of mixed effects modelling assessing the effects of the presence of uni- or bilateral thyroid adenoma and study timepoint on TT4, TSH, and creatinine concentration are shown in Table 4. The effect of the study timepoint on TT4, TSH, and creatinine concentration is also shown in Figure 4.

The presence of uni- or bilateral thyroid adenoma had an effect on TSH concentration (*p* = 0.034). Cats with bilateral adenomas had significantly higher TSH concentrations than cats with unilateral adenomas. The study timepoint had a significant effect on TT4 (*p* < 0.001), TSH (*p* < 0.001), and creatinine (*p* < 0.001) concentration. Total thyroxine concentration decreased significantly at week 1 and further at month 1, meaning that TT4 was significantly higher at enrolment when compared to all study timepoints (all *p* < 0.001) and TT4 was also significantly higher at week 1 in comparison to all other study timepoints (all *p* < 0.001) apart from enrolment. Total thyroxine concentration did not change significantly following month-1 re-examination. Thyrotropin and creatinine concentration increased significantly at month 1, meaning that both TSH and creatinine concentration were significantly lower at enrolment when compared to all study timepoints (all *p* < 0.001) but week 1 (for TSH: *p* = 0.64; for creatinine: *p* = 0.86) and at week 1 when compared to all study timepoints (all *p* < 0.001) but enrolment. There was no significant increase in TSH or creatinine concentration following month-1 re-examination post RAIT.

#### Cats with and without Increase in TSH Concentration above Reference Range

Of the 42 cats with uni- or bilateral adenoma and the resolution of hyperthyroidism, a TSH increase above the RR was detected at some timepoint during this study in 28/42 (67%) cats, while TSH remained below the upper limit of the RR in 13 cats (13/42, 31%). In 1/42 (2%) cats, only the results of week-1 re-examination were available and because the mixed effects modelling showed a significant increase in TSH concentration up to month 1 following RAIT, this cat was excluded from the analysis.

**Figure 5:**
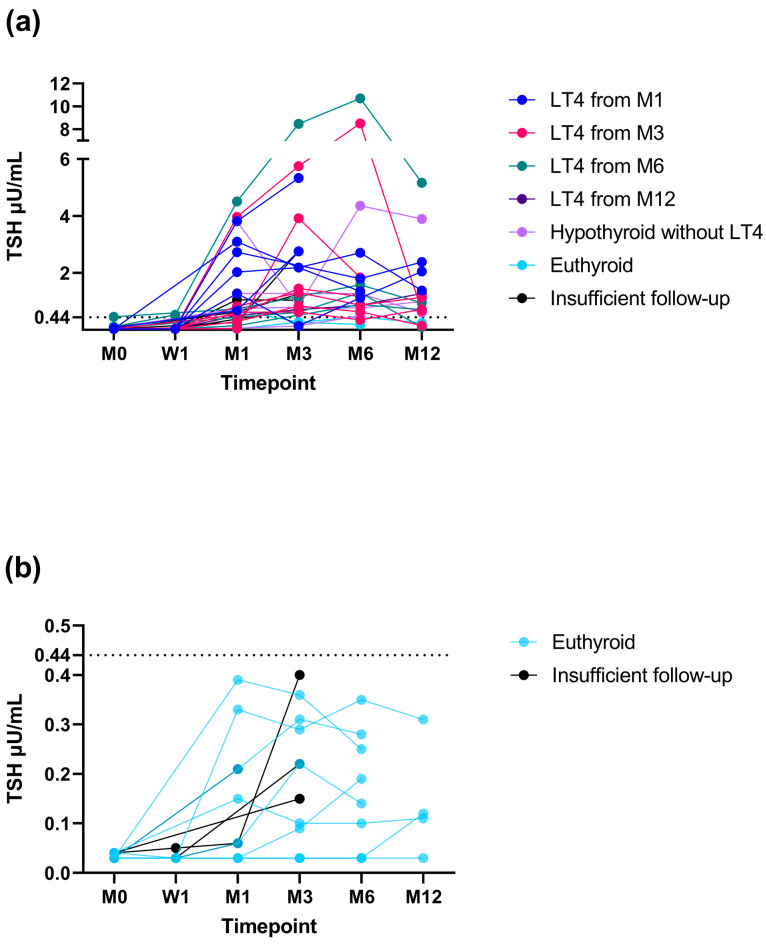
Changes in thyrotropin (TSH) concentration in 28 cats that did (**a**) and 13 cats that did not (**b**) experience a TSH increase above the reference range (RR) during this study. Only cats that had a minimum of a one-month follow-up were included in the graphs. Hypothyroid cats are colour-coded depending on the study timepoint when levothyroxine (LT4) replacement was started (month 1 [M1], dark blue; month 3 [M3], red; month 6 [M6], green; month 12 [M12], dark violet). Hypothyroid cats without LT4 replacement are in light violet, euthyroid cats in light blue, and cats with an insufficient follow-up to differentiate between eu- and hypothyroidism in black. The upper limit of the RR for TSH (0.44 µU/mL) is marked as a dotted horizontal line. Legend: W, week.

Of the 28 cats that experienced a TSH increase above the RR, 2/28 (7%) became euthyroid, 21/28 (75%) became hypothyroid, and 5/28 (18%) had an insufficient follow-up to differentiate between eu- and hypothyroidism. Of the 13 cats that did not experience a TSH increase above the RR during this study, 8/13 (62%) became euthyroid and 5/13 (38%) had an insufficient follow-up to differentiate between eu- and hypothyroidism. The changes in TSH concentration over time in cats that did and cats that did not experience a TSH increase above the RR are depicted in Figure 5. Of the 20 cats with TSH results available at month-12 re-examination, in 12/20 (60%), TSH was still increased >RR (9/12 cats with increased TSH were receiving LT4 supplementation).

To assess whether any variables were associated with a TSH increase above the RR, the TT4 concentration prior to RAIT, proportion of cats with uni- or bilateral disease, and radioiodine dose were compared between cats that experienced a TSH increase >RR and those that did not (Table 5). Cats that experienced a TSH increase >RR more frequently suffered from bilateral disease, but there was no difference in the initial TT4 prior to RAIT or the iodine dose administered to treat hyperthyroidism.

## 4. Discussion

This study revealed that TT4 concentration significantly decreases already one week following RAIT and a further decrease is seen one month later, but no significant changes occurred afterwards. Furthermore, TSH and creatinine concentration also significantly increased one month post RAIT (no significant change at one week post therapy), with no significant changes later on. However, in individual cats, hypothyroidism and an increase in creatinine concentration might be diagnosed as late as 12 months post RAIT. Furthermore, cats with bilateral adenomas had higher TSH concentrations throughout this study when compared to cats with unilateral disease.

The finding of significant reduction in TT4 concentration within the first month following RAIT is in agreement with a previous study that reported that most cats become euthyroid within 2 weeks of therapy [6]. Changes in TSH concentration over time have not been directly evaluated in previous studies, although the authors measured TSH to assess for possible hypothyroidism following RAIT. In a study by Lucy et al. comparing the efficacy of a low and a standard RAI dose, it is obvious from the graphical presentation of the results that median TSH concentration increased one month post RAIT and remained increased at three and six months, but statistical comparisons between study timepoints were not made [12]. Although direct comparisons between ours and Lucy’s study cannot be made, in both studies, increased TSH concentrations were detected one month following RAIT and persisted at later re-examination timepoints. In our study, TSH was still significantly higher 12 months following RAIT when compared to enrolment (i.e., hyperthyroid state). Interestingly, in 12/20 cats with samples available at the month-12 re-examination, TSH concentration still was >RR, even though 9/12 of these cats were receiving LT4 replacement at this timepoint (all 9 had been receiving LT4 for a minimum of 6 months). Of the three cats that had increased TSH but were not receiving LT4 replacement at month 12, one cat was started on LT4 replacement at this timepoint because of a newly identified increase in creatinine concentration > 140 µmol/L, but two cats had creatinine < 140 µmol/L and were therefore not treated (they also had no clinical signs of hypothyroidism). Because no longer follow-up was available in our study, we are unaware if these cats later developed an increase in creatinine concentration or clinical signs of hypothyroidism and needed LT4 replacement or their TSH concentrations normalised. The finding of long persistence of an increased TSH concentration, however, raises the question if LT4 replacement is only indicated in azotaemic cats or those with clinical signs as is the current practice [26], or if there is a need for change in treatment strategy. A longer follow-up and survival analysis would be needed to answer this question, but a previous study that instituted LT4 replacement in a similar fashion to ours (azotaemic or clinically hypothyroid cats) found longer survival in cats treated with levothyroxine [7]. In that study, however, the TSH normalised following LT4 replacement in 18/19 cats with iatrogenic hypothyroidism post RAIT (all cats were receiving supplementation for at least one month, but exact time of TSH re-check was not reported). On the other hand, in six hypothyroid cats treated with levothyroxine in another investigation, TSH did not normalise within the timeframe of the study (six months) [12], similar to our experience. It cannot be said if the persistence of the TSH increase in our cats indicates undersupplementation, but the reduction in creatinine concentration to <140 μmol/L in 8/15 hypothyroid cats with an increased creatinine concentration and sufficient follow-up (at least 6–12 months post RAIT) in our study indicates that at least in terms of the kidney function, the supplementation was sufficient. Nevertheless, our findings indicate that the follow-up of RAI-treated cats for longer than six months is needed.

Interestingly, although TSH concentration increased above the RR in most (28/42, 67%) of the cats with the resolution of hyperthyroidism in our study, 13/42 (31%) did not experience a TSH increase above the RR. The reason for this finding is unknown, but at least in some cats, where no change in TSH concentration was detected at all (i.e., TSH remained suppressed), a possible reason might be the low sensitivity of the human TSH assay used in this study to detect feline TSH. Low sensitivity to detect feline TSH has also been reported for the canine TSH assay [28]. Therefore, future studies using the newly developed TSH assay that is able to detect very low concentrations of feline TSH might be able to detect TSH changes in a range that is currently under the lower detection limit of the human TSH assay used in this study [20], or the canine assay used in other studies [28]. Interestingly, cats that showed a TSH increase above the RR were more likely to suffer from bilateral disease than those who did not show a TSH increase. Similarly, mixed effects modelling revealed that cats with bilateral disease had higher TSH concentrations throughout this study than cats with unilateral disease. A previous study revealed that cats with bilateral hyperthyroidism are at higher risk of developing hypothyroidism post RAIT [19]. In another study, cats that received a higher (i.e., standard) iodine dose (4 mCi) had higher TSH concentrations at one, three, and six months post RAIT than cats treated with a lower dose (2 mCi) [12]. The authors did not specifically discuss this finding, but they concluded that a lower iodine dose (2 mCi) was safe and effective and resulted in a lower incidence of hypothyroidism. However, that study only included cats with mild-to-moderate hyperthyroidism (T4 < 13 μg/dL) and is therefore not directly comparable to our study (median TT4 concentration prior to RAIT in cats with adenomas that achieved resolution of hyperthyroidism in our study was 12.9 μg/dL; only 21/42 [50%] of these cats had TT4 < 13 μg/dL). In our study, there was no significant difference in the median administered iodine dose between cats that did (4.1 mCi) and those that did not (2.6 mCi) experience a TSH increase, making a potential overtreatment an unlikely reason for the variability in the occurrence of the TSH response.

Our study also evaluated changes in creatinine concentration and found a significant increase at one month post RAIT but no significant increase afterwards. A total of 11 cats (11/37) with available creatinine measurement had an increase in creatinine concentration > 140 µmol/L at month-1 re-examination and overall, 28/42 (67%) cats with the resolution of hyperthyroidism had an increase in creatinine concentration > 140 µmol/L at some point during this study (although the creatinine concentration dropped < 140 µmol/L in 10/22 with a sufficient follow-up, discussed below). Previous studies have shown that GFR declines one month following RAIT, but cats usually become azotaemic after 3–6 months of the treatment [10,11]. However, in one of those studies, upper RR of creatinine concentration was higher than the IRIS cut-off of 140 µmol/L (at least based on the graphical presentation of the results in that publication; no clear cut-off was reported by the authors) [10]. Therefore, some cats might have been azotaemic at month 1 post RAIT in that study if the IRIS cut-off for azotaemic CKD had been applied. In the second study, the IRIS cut-off of 140 µmol/L was used to identify azotaemia [11]. Five cats (5/21, 24%) were diagnosed with impaired kidney function within six months post RAIT (based on azotaemia and reduced GFR), some of which became azotaemic at month-1 re-examination (number not specified in publication). In those five cats with impaired kidney function, the creatinine concentration did not change significantly following month-1 re-examination and all five cats were azotaemic 3 months post RAIT. In other larger studies, which, however, did not measure GFR, azotaemia was detected in 42/262 (16%) [13], 263/1400 (19%) [16], 158/655 (24%) [14], and 62/189 (33%) cats at 6 to 12 months post RAIT [12]. Although this seems lower than in our study, these larger studies again used a higher creatinine cut-off (2 mg/dL, ~176.8 μmol/L). Therefore, the frequencies of azotaemia in those reports are not directly comparable to our study. Furthermore, those other studies mainly assessed the proportion of azotaemic cats in a cross-sectional fashion (at 6 or 12 months post RAIT) rather than longitudinally as in our study. In our study, a diagnosis of azotaemic CKD was only made in cats with a persistent increase in creatinine concentration (creatinine concentration repeatedly ≥140 µmol/L) that were followed-up for a minimum of six months post RAIT. Based on this criterion, azotaemic CKD was diagnosed in 11 cats (11/42, 26% of cats with uni-/bilateral disease and resolution of hyperthyroidism). The documentation of persistence of an increase in creatinine concentration prior to making a diagnosis of CKD is highly important in RAI-treated cats because of the association of azotaemia with iatrogenic hypothyroidism and possible resolution of azotaemia with LT4 replacement identified in previous studies (discussed below). It is also important to understand that although CKD could only be diagnosed following RAIT and the resolution of hyperthyroidism, at least some of those 11 cats with azotaemic CKD in our study likely suffered from non-azotaemic CKD prior to RAIT. In a hyperthyroid state, a diagnosis of CKD might be difficult to make, not only because of the increased GFR [29,30], but also because some indicators of CKD such as reduced urine concentrating ability [14], or proteinuria might be present in hyperthyroid cats and not necessarily indicate the presence of CKD [11]. On the other hand, cats that only developed an increased creatinine concentration at later timepoints following RAIT (i.e., after six months) and were diagnosed with CKD later on might not have had masked CKD prior to RAIT but developed “new” CKD as this is a common condition in older cats.

In our study, 22/28 cats that developed an increase in creatinine concentration at some point following RAIT had a follow-up of at least 6 months post RAIT, and creatinine concentration dropped <140 μmol/L in 10/22 (46%) of these cats (including 8 cats on LT4 replacement). The resolution of azotaemia in hypothyroid cats on LT4 supplementation post RAIT has been reported in 4/19 cats in another study and the authors indicated improvement in the remaining 15/19 cats, too [7]. Similarly, azotaemia resolved in 7/14 cats with iatrogenic hypothyroidism secondary to treatment with antithyroid drugs following dose reduction and the restoration of euthyroidism [31]. Generally, across all studies (concerning mainly RAI-treated cats), azotaemia was more prevalent in cats with iatrogenic hypothyroidism than in cats that achieved euthyroidism post treatment [13,14,16]. Given the shorter survival of azotaemic hypothyroid cats when compared to non-azotaemic hypothyroid cats in a study of cats treated either with antithyroid drugs or thyroidectomy [32], and longer survival in azotaemic hypothyroid cats post RAIT that received LT4 replacement [7], the early institution of LT4 replacement in cats with iatrogenic hypothyroidism seems warranted. Some authors suggest that LT4 replacement might offer a survival benefit in non-azotaemic cats with iatrogenic hypothyroidism too [33], although that study has, to the authors’ knowledge, not yet been published in full. Given the possible positive effects of the treatment of iatrogenic hypothyroidism on survival [7], our institution is using a creatinine cut-off of 140 μmol/L rather than a laboratory-own creatinine cut-off to allow for the early institution of LT4 replacement in RAI-treated cats.

Although this study did not aim to compare the treatment outcome to other published studies, the treatment failure of 9% (4/46 cats with adenomas) might seem high at first sight, given the results of large studies generally reporting treatment failure of <5% [12,13,16,17]. However, there are other smaller studies that reported treatment failures of 19 or 23% [34], and 9 or 16% (each study compared two treatment protocols and is therefore reporting two failure rates) [35], respectively. Furthermore, some of those reported large studies specifically only included cats with mild-to-moderate hyperthyroidism [12]. On the other hand, the hypothyroidism rate in our study (21/42, 50%) seems high but is comparable to cats treated with the 4 mCi-fixed dose (25/39, 64%) in Lucy’s study [12], as well as to a report of 55 cats treated with RAIT using an individualised scoring system (29/55, 53%) [36]. In those two latter studies, the proportion of hypothyroid cats was evaluated at six [12] or six to nine months post RAIT [36], and it is therefore unknown if some might have developed hypothyroidism later on.

The main limitation of our study is that cats did not present to all scheduled re-examinations and a sufficient follow-up (at least six months post RAIT) was only available for 29/42 cats with uni-/bilateral disease and the resolution of hyperthyroidism. This was mainly due to financial reasons, but the cats’ temperament also played a role. Furthermore, a number of cats presented during the study period could not be enrolled for several reasons, the most common being lack of owner consent with study participation, which reduced our sample size and therefore some analyses might be underpowered. Also, hypothyroidism was diagnosed based on TT4 and TSH measurement and not by thyroid scintigraphy, which is the gold standard in assessment of thyroid function. Thyroid scintigraphy, however, requires sedation and was therefore considered fairly invasive when compared to TSH measurement, which in one study was shown to be a better parameter than TT4 to differentiate between hypothyroid and euthyroid cats in comparison to the gold standard evaluation of thyroid function by scintigraphy [7]. Furthermore, LT4 replacement was started fairly early in some cats (in six cats as early as one month following RAIT) and apart from one cat, no attempts were made to taper the dose and discontinue the treatment (in this one cat, discontinuation of treatment was attempted on owner request, but hypothyroidism was confirmed after LT4 replacement was stopped and it therefore was re-started). Therefore, whether some cats might have suffered from transient hypothyroidism could not be clarified in our study. In a small study that followed RAI-treated cats for a period of one year, however, no cats developed transient overt or transient subclinical hypothyroidism before they became euthyroid [37]. We have decided against the tapering and discontinuation of LT4 because a number of cats still had increased TSH at the final month-12 re-examination, despite TT4 being within a desired range (2.5–3.5 µg/dL). Furthermore, GFR was not measured, and creatinine was the only parameter of renal function assessed in this study. But the assessment of GFR requires several blood samples and was therefore not included in our study protocol as we were concerned about owner compliance and lack of consent. Since owners usually travel long distances to our institution for their cat’s RAIT, all re-examinations took place at the referring veterinarians and therefore follow-up GFR measurement would not have been feasible. On the other hand, measurement of SDMA and assessment of USG, proteinuria, and blood pressure would all have been useful, non-invasive, and desirable tests. However, although these were performed in some cats, they were not assessed in any standardised manner and in most cats, these data were not available and were therefore not included in the analysis. Assessment of USG might be helpful to differentiate between pre-renal and renal azotaemia; however, some cats with CKD might retain renal concentrating ability; therefore, the presence of kidney disease cannot be ruled out based on USG > 1035 [38,39]. Furthermore, due to radiation safety measures, urine samples could only be collected and examined from month-3 examination onwards. Also, measurement of TSH should ideally be performed using an assay that is able to detect very low concentrations of feline TSH [28], rather than the human assay used in this study [20], or canine assay used in other studies [12,40]. Finally, the sensitivity and specificity of the combination of low or low-normal TT4 and increased TSH to detect hypothyroidism using the human TSH assay have not been evaluated. Increased TSH concentration was a better parameter than TT4 or free T4 to identify iatrogenic hypothyroidism when compared to the gold standard evaluation of thyroid function by scintigraphy [7], but in that study, a canine TSH assay was used.

## 5. Conclusions

This study revealed that TT4 concentration significantly decreases already one week following RAIT, with a further decrease one month post treatment, but no significant changes occurred afterwards. Furthermore, TSH and creatinine concentration also significantly increase one month post RAIT, with no significant changes later on. Although an increase in creatinine concentration and hypothyroidism can be identified as early as one month post RAIT in some cases, in a substantial proportion of cats, these conditions were diagnosed at later timepoints and in some cats even as late as 12 months post RAIT. The majority of cats will show an increase in TSH concentration above the RR post RAIT, which is more common in cats with bilateral hyperthyroidism. Cats with bilateral hyperthyroidism have also been found to have higher TSH concentrations following RAIT by mixed effects modelling.

## Figures and Tables

**Table 1:** The median (range, interquartile range [IQR]) concentration of total thyroxine (TT4), thyrotropin (TSH), and creatinine at each study timepoint and the number of cats (n) for which these test results were available. Only test results of cats with uni- or bilateral adenoma in which hyperthyroidism resolved (eu- or hypothyroid) were included. Cats with suspected carcinoma and cats that remained hyperthyroid were excluded.

Timepoint	TT4 (μg/dL) Median (Range, IQR) n (%)	TSH (µU/mL) Median (Range, IQR) n (%)	Creatinine (μmol/L) Median (Range, IQR) n (%)
Enrolment	12.9 (6.0–41.4, 9.7–25.3) 42/42 (100%)	0.03 (0.03–0.46, 0.03–0.04) 42/42 (100%)	84 (50–139, 72–98) 42/42 (100%)
Week 1	4.5 (1.8–9.9, 3.4–6.2) 31/42 (74%)	0.03 (0.03–0.53, 0.03–0.03) 26/42 (62%)	85 (61–136, 76–95) 35/42 (83%)
Month 1	1.3 (0.5–3.7, 0.6–2.0) 38/42 (90%)	0.52 (0.03–4.51, 0.15–1.00) 38/42 (90%)	124 (78–222, 109–146) 37/42 (88%)
Month 3	1.6 (0.5–3.6, 0.5–1.9) 35/42 (83%)	0.71 (0.03–8.46, 0.25–1.38) 35/42 (83%)	137 (73–209, 121–150) 35/42 (83%)
Month 6	1.6 (0.5–3.7, 1.2–2.3) 29/42 (69%)	0.86 (0.03–10.70, 0.28–1.35) 29/42 (69%)	137 (86–237, 121–152) 28/42 (67%)
Month 12	2.2 (0.9–6.5, 1.8–2.9) 20/42 (48%)	0.72 (0.03–5.17, 0.22–1.30) 20/42 (48%)	144 (82–221, 118–168) 20/42 (48%)

**Table 2:** Number of cats (n) with uni-/bilateral adenoma that became euthyroid, became hypothyroid (including cats with and without levothyroxine (LT4) replacement), or did not have sufficient follow-up to differentiate between euthyroidism and hypothyroidism.

	Euthyroid Cats (n = 10)	All Hypothyroid Cats (n = 21)	Hypothyroid Cats with LT4 Replacement (n = 17)	Hypothyroid Cats without LT4 Replacement (n = 4)	Cats with Insufficient Follow-Up (n = 11)
Unilateral adenoma n (%)	6 (60%)	7 (33%)	6 (35%)	1 (25%)	5 (45%)
Bilateral adenoma n (%)	4 (40%)	14 (67%)	11 (65%)	3 (75%)	6 (55%)

**Table 3:** The median (range, interquartile range [IQR]) concentration of total thyroxine (TT4) and thyrotropin (TSH) at each study timepoint and the number of cats (n) for which these test results were available, after removing test results of cats under levothyroxine (LT4) replacement (i.e., 6 cats were receiving LT4 replacement at month 3, 12 at month 6, and 16 at month 12). Only cats with uni- or bilateral adenoma that became eu- or hypothyroid post radioiodine treatment (RAIT) were included. Cats with suspected carcinoma were excluded.

Timepoint	TT4 (μg/dL) Median (Range, IQR) n (%)	TSH (µU/mL) Median (Range, IQR) n (%)
Enrolment	12.9 (6.0–41.4, 9.7–25.3) 42/42 (100%)	0.03 (0.03–0.46, 0.03–0.04) 42/42 (100%)
Week 1	4.5 (1.8–9.9, 3.4–6.2) 31/42 (74%)	0.03 (0.03–0.53, 0.03–0.03) 26/42 (62%)
Month 1	1.3 (0.5–3.7, 0.6–2.0) 38/42 (91%)	0.52 (0.03–4.51, 0.15–1.00) 38/42 (91%)
Month 3	1.5 (0.5–3.0, 0.5–1.9) 30/36 (83%)	0.63 (0.03–8.46, 0.23–1.14) 30/36 (83%)
Month 6	1.6 (0.5–3.7, 1.2–2.1) 19/30 (63%)	0.46 (0.03–10.70, 0.19–1.08) 19/30 (63%)
Month 12	1.8 (0.9–2.7, 1.7–2.1) 9/26 (35%)	0.31 (0.03–3.90, 0.12–1.00) 9/26 (35%)

**Table 4:** The *p*-values from mixed effects modelling assessing the effects of the presence of uni- or bilateral thyroid adenoma and study timepoint (enrolment and week 1, month 1, month 3, month 6, and month 12 following radioiodine treatment [RAIT]) on total thyroxine (TT4), thyrotropin (TSH), and creatinine concentration in 42 cats with uni-/bilateral adenoma and the resolution of hyperthyroidism following RAIT. In cats receiving levothyroxine (LT4) replacement, the TT4 and TSH results from re-examinations following the LT4 treatment start were excluded from the analysis. Significant *p*-values are in bold.

Variable	Study Timepoint	The Presence of Uni- or Bilateral Thyroid Adenoma
Log (TT4 + 1)	**<0.001**	0.1
√TSH	**<0.001**	**0.034**
Creatinine	**<0.001**	0.83

**Table 5:** Comparison of total thyroxine (TT4) concentration prior to radioiodine treatment (RAIT) and number (n) of cats with uni- or bilateral disease and radioiodine dose between cats that experienced thyrotropin (TSH) increase above the reference range (RR) at some timepoint following RAIT (n = 28) and those that did not (n = 13). Only cats with uni-/bilateral adenoma and resolution of hyperthyroidism and minimum of one-month follow-up were included in analysis. Significant *p*-values are in bold.

	Cats with TSH Increase (n = 28)	Cats without TSH Increase (n = 13)	*p*-Value
TT4 [μg/dL] at enrolment: median (range, IQR)	14.2 (6.0–41.4, 10.3–28.6)	11.0 (6.0–40.5, 9.0–15.9)	0.135
Unilateral disease Bilateral disease	n = 8 n = 20	n = 10 n = 3	**0.006**
Radioiodine dose (mCi): median (range, IQR)	4.1 (1.9–5.9, 3.5–5.0)	2.6 (1.5–5.7, 2.0–4.7)	0.114

Legend: IQR, interquartile range.

## Data Availability

The data presented in this study are available on request from the corresponding author.

## Supplementary Materials

The following supporting information can be downloaded at: https://www.mdpi.com/article/10.3390/ani14182627/s1, File S1: Information on validation of chemiluminescence assay for measurement of human TSH (ADVIA Centaur, Siemens Healthineers, Erlangen, Germany) (based on reference no. [20]); Report of the European Veterinary Endocrine Quality Assurance Scheme on measurement of feline TSH using the human TSH assay at the laboratory Biocontrol (Ingelheim, Germany); Reasons of the 30/81 presented during the study period for not being enrolled into the study; Information about Technetium Uptake (TcTU), homogeneity of TcTU and thyroid volume of the 50 cats treated with radioiodine.
