# Supplementary material for: Effect of Presence of Uni- or Bilateral Thyroid Adenoma on Recovery of Pituitary–Thyroid Axis and Creatinine Concentration in Radioiodine-Treated Cats
Source: Animals (Basel). 2024 Sep 10;14(18):2627. doi: 10.3390/ani14182627 (PMC11428651; doi:10.3390/ani14182627)
Supplement: Supplementary file 1 [file animals-14-02627-s001.zip › animals-3136678-supplementary.pdf]

1. Validation of chemiluminescence assay for measurement of human TSH (ADVIA Centaur, Siemens Healthineers, Erlangen, Germany)

Publication: M. Puille, D. Auch, T. Spillman, L. Birke, and R. Bauer, "Bestimmung von TSH und freien Schilddrüsenhormonen in der Hyperthyreosediagnostik der Katze," *Tierarztl. Prax. Ausgabe K Kleintiere - Heimtiere*, vol. 28, pp. 289–94, 2000.

Sandwich immunoassay using polyclonal anti-human TSH antibodies (ovine)

Inter-assay precision: pooled serum of 27 euthyroid cats, assessed on 10 consecutive days, inter-assay CV: 2.7%

Intra-assay precision: serum of 5 cats with different TSH concentrations, intra-assay CV: 4.3 ± 1.5%

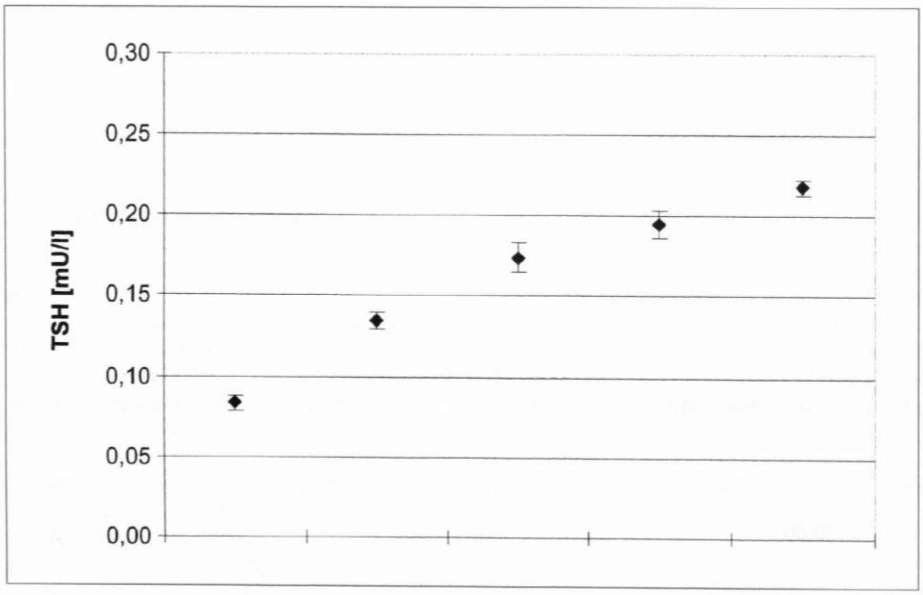

Abb. 3 Ergebnis der Mehrfachbestimmung (Intraassaypräzision) von TSH im Serum fünf unterschiedlicher Katzen (Mittelwert und Standardabweichung)

Lower limit of detection: 0.015 μU/L

Reference interval: 59 clinically healthy cats aged 2-5 years, TSH concentration: 0.24 ± 0.2 μU/L

TSH concentration in 10 hyperthyroid cats, with clinical signs and laboratory evidence of hyperthyroidism (based on TT4 and fT4 measurement) in all cats and scintigraphy in 7 cats: 0.02 ± 0.05 μU/L

European Veterinary Endocrine Quality Assurance Scheme (data for measurement of feline TSH using the human assay – Method 4)

ESVE Veterinary Endocrinology External Quality Assessment Scheme
PARTICIPANT REPORT

Lab ID
A730

Release Month:	Nov-19
Release Number:	015
Species:	Feline

This Release

TSH

	n	Mean	StDev	%CV
Method 1	44	0.11	0.02	15.5
Method 2	14	0.09	0.01	14.8
Method 3	2	0.08	0.01	6.9
**Method 4**	**2**	**0.10**	**0.02**	**22.3**
Method 5	1	0.13		
Method 6	1	0.03		
Method 7	1	0.13		
Method 8	0			
Method 9	0			
Method 10	0			
Method 11	0			
Method 12	0			
		Trimmed	*Adjusted*	
All Methods	65	0.11	0.020	18.2

		SD multiple	Classification	TEa (BV)
Your result	0.11	0.00	<2SD	Optimum
Your 2nd result	NONE		NONE	

Your Method: Method 4 ADVIA Centaur TSH

Your 2nd Method:

Note: Reported results ranged from below the limit of detection (<0.25ng/ml) to 0.14ng/ml.
Methods 1,2 and 3 represent the same manufacturer's chemiluminescent assay on 3 platforms (Siemens Immulite). Methods 5 and 7 were in-clinic analysers. Method 4 is a human method

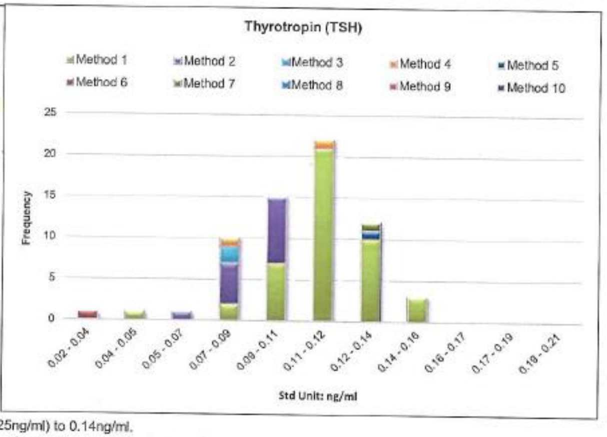

6-Cycle History

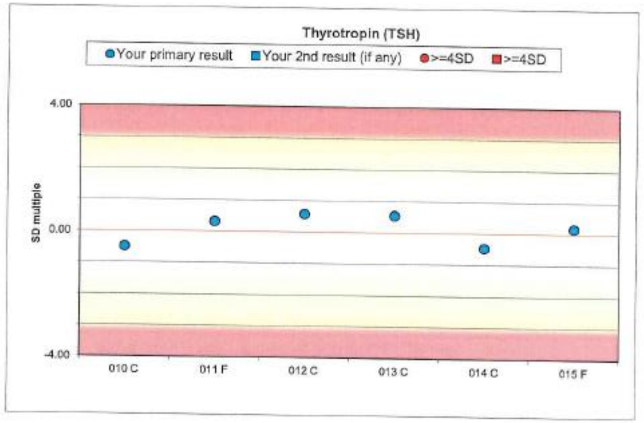

ESVE Veterinary Endocrinology External Quality Assessment Scheme
PARTICIPANT REPORT

Lab ID
A730

This Release

Release Month:	Nov-19
Release Number:	015
Species:	Feline

TSH

As has been the case in previous releases, the heavy reliance among labs on automated platforms from a single supplier contributes to all-method CV. As has also been the case with previous feline releases, our CV% are not as good as with canine samples which is not unexpected as the methods used are mostly branded as "canine" methods. There is varaibility among methods in their transferability to feline samples. For example, Method 6 appears optimised to canine TSH to the extent that it generates lower results than other methods when presented with a feline sample. Method 4 is a human method whose results have been converted to ng/ml from uIU/ml using data from an unpublished method comparison study.

2. Reasons of the 30/81 cats for not being enrolled into the study

The owners of 13 cats did not give their consent for participation in the study. In six cats, radioiodine treatment (RAIT) was not performed because the cat died prior to RAIT (n=3), diseases other than hyperthyroidism (e.g., neoplasia) carrying poor prognosis were identified (n=2) or no abnormal thyroid tissue was detectable on scintigraphy (i.e., the cat was euthyroid) (n=1). Five cats were not enrolled due to preexisting azotaemia (creatinine concentration ≥ 140 µmol/l) and two because of abnormal kidney morphology. Two cats did not receive an ultrasound of the kidneys prior to RAIT due to organisation problems and because this was the prerequisite for enrolment onto the other study, they could not be enrolled in this study either. Finally, two cats were not enrolled because they lived abroad and there were concerns about sending samples with the post and analyte stability.

3. Information about Technetium Uptake (TcTU), homogeneity of TcTU and thyroid volume of the 50 cats treated with radioiodine

Cat number	Technetium uptake (%)	Homogeneity	Thyroid volume (ml)
1	28.5	Heterogeneous	
2	6.6	Homogeneous	5.7
3	2.1	Homogeneous	
4	9.6	Homogeneous	2.8
5	6.7	Heterogeneous	3.3
6	2.3	Homogeneous	0.7
7	16.0	Homogeneous	3.6
8	1.4	Homogeneous	0.7
9	1.7	Homogeneous	1.6
10	3.5	Homogeneous	2.8
11	5.0	Homogeneous	
12	6.9	Homogeneous	1.6
13	5.0	Homogeneous	1.1
14	5.5	Homogeneous	1.1
15	2.4	Homogeneous	0.6
16	15.2	Homogeneous	3.5
17	14.9	Homogeneous	2.4
18	16.6	Homogeneous	
19	12.8	Homogeneous	
20	6.0	Homogeneous	4.0
21	32.0	Homogeneous	
22	5.0	Homogeneous	
23	21.4	Homogeneous	4.3
24	5.2	Homogeneous	1.5
25	3.0	Homogeneous	
26	16.0	Homogeneous	1.8
27	20.5	Homogeneous	2.2
28	6.8	Homogeneous	
29	2.0	Homogeneous	0.9
30	7.0	Homogeneous	
31	9.2	Homogeneous	2.5
32	2.1	Homogeneous	1.3
33	8.3	Homogeneous	4.0
34	2.9	Homogeneous	0.7
35	4.8	Homogeneous	1.7
36	8.5	Homogeneous	1.5
37	2.9	Homogeneous	3.2
38	2.8	Homogeneous	0.7
39	12.7	Homogeneous	2.7
40	11.7	Homogeneous	5.5
41	3.4	Homogeneous	0.7
42	11.9	Homogeneous	2.0
43	6.4	Homogeneous	1.8
44	6.6	Homogeneous	
45	14.9	Heterogeneous	
46	42.2	Heterogeneous	
47	8.0	Homogeneous	4.7
48	15.4	Homogeneous	6.5
49	17.0	Homogeneous	5.0
50	1.3	Homogeneous	0.8
